# Comparing the economic terms of biotechnology licenses from academic institutions with those between commercial firms

**DOI:** 10.1371/journal.pone.0283887

**Published:** 2023-03-31

**Authors:** Prateet Shah, Gregory Vaughan, Fred D. Ledley

**Affiliations:** 1 Center for Integration of Science and Industry, Bentley University, Waltham, Massachusetts, United States of America; 2 Department of Mathematical Sciences, Bentley University, Waltham, Massachusetts, United States of America; 3 Department of Natural & Applied Sciences, Bentley University, Waltham, Massachusetts, United States of America; 4 Department of Management, Bentley University, Waltham, Massachusetts, United States of America; Sichuan Agricultural University, CHINA

## Abstract

Licenses of drug-related biotechnologies from academic institutions to commercial firms are intended to promote practical applications of public sector research and a return on government investments in biomedical science. This empirical study compares the economic terms of 239 biotechnology licenses from academic institutions to biotechnology companies with 916 comparable licenses between commercial firms. Academic licenses had lower effective royalty rates (median 3% versus 8%, p<0.001), deal size (median $0.9M versus $31.0M, p<0.001), and precommercial payments (median $1.1M versus $25.4M, p<0.001) than corporate licenses. Controlling for the clinical phase of the most advanced product included in the license reduced the median difference in effective royalty rate between academic and corporate licenses from 5% (95% CI 4.3–5.7) to 3% (95% C.I. 2.4–3.6) but did not change the difference in deal size or precommercial payments. Excluding licenses for co-commercialization did not change the effective royalty rate but reduced the median difference in deal size from $15.8M (95% CI 14.9–16.6) to $11.4M (95% CI 10.4–12.3) and precommercial payments from $9.0M (95% CI 8.0–10.0) to $7.6M (95% CI 6.8–8.4). Controlling for deal terms including exclusivity, equity, or R&D in multivariable regression had no substantive effect on the difference in economic terms. This analysis suggests the economic returns associated with biotechnology licenses from academic institutions are systematically lower than licenses between commercial firms and that this difference is only partially accounted for by differences in the intrinsic terms of the license agreements. These results are discussed in the context of a reasonable royalty rate, recognizing that factors extrinsic to the license agreement may reasonably impact the negotiated value of the license, as well as economic theories that view government as an early investor in innovation and technology licenses as a mechanism for achieving a return on investment.

## Introduction

The licensing of biotechnologies from academic institutions to industry has a central role in pharmaceutical innovation. These licenses provide a mechanism for transferring the scientific insights and technological advances generated by government-funded research to the biopharmaceutical industry for development and commercialization. The economic terms of these licenses also represent the primary channel by which the public sector receives an economic return on government investments in biomedical science that contributes to new pharmaceutical products.

This study examines the economic returns from biotechnology licenses from academic institutions to industry (“academic licenses”) compared to those between for-profit corporate entities (“corporate licenses”). Previous studies have observed that the economic returns from academic licenses are less favorable than those from corporate licenses [1–5]. The present work extends those observations by asking whether this disparity is associated with differences in the intrinsic terms of the license agreements.

Specifically, this study compares the effective royalty rate, precommercial payments, and total deal value of academic licenses of drug-related biotechnologies with those of corporate licenses. The analysis then asks whether disparities in these economic terms are associated with differences in the licensee (biotech or pharma) or intrinsic terms of these licenses including the development stage of products covered by the license agreement, the activities anticipated under the agreement (research, development, or co-commercialization), exclusive or non-exclusive licensing, or the inclusion of equity in the agreement.

The results show that disparities in economic returns from academic and corporate licenses are only partially associated with the intrinsic terms of the license agreements. These results are considered in the context of the legal concept of a reasonable royalty rate, which recognizes that extrinsic as well as intrinsic factors may reasonably impact the negotiated value of a license. These results are also discussed in the context of economic theories that recognize government as an early-stage investor in pharmaceutical innovation and the licensing of academic biotechnologies to industry as a means for achieving a social return on public sector investments in biomedical science.

### Background information

#### Technology licensing in pharmaceutical innovation

US science policy since the 1950s has was greatly influenced by the model of innovation articulated by Vannevar Bush in the landmark report The Endless Frontier [6–9]. This report recognized that basic science had a foundational role in technological innovation, writing: “*Basic research leads to new knowledge*. *It provides scientific capital*. *It creates the fund from which the practical applications of knowledge must be drawn*” [6]. The report also localized basic research in colleges and universities, writing: “*Publicly and privately supported colleges and universities and the endowed research institutes must furnish both the new scientific knowledge and the trained research workers*. *These institutions are uniquely qualified by tradition and by their special characteristics to carry on basic research*” [6]. In contrast, the report localized product development in industry, writing “*Industry will fully rise to the challenge of applying new knowledge to new products*. *The commercial incentive can be relied upon for that*” [6]. To facilitate innovation, The Endless Frontier recommended that “…*basic research should be strengthened by use of public funds*” provided to colleges, universities, and research institutes, and that the government should “*devise and promote the use of methods of improving the transition between research and its practical application in industry”* [6].

Contemporary, targeted drug discovery typically begins with basic research describing the mechanisms of health and disease and potential targets for drug discovery, which leads to applied research focused on target validation and lead discovery [8,10–13]. This research takes place primarily in academic or government laboratories with public-sector funding [14,15]. In the US, most of this funding comes from the National Institutes of Health (NIH).

Cleary et al. have described the NIH investment in research associated with the 356 drugs approved by the FDA from 2010–2019 [8,13]. These studies identified $187 billion in NIH funding for published research related to these products, of which $156 billion represented basic research on the drug’s biological target and $31 billion represented applied research on the drugs themselves [8]. These studies also show that the NIH invests approximately $800 million for basic and applied research related to each first-in-class drug prior to approval [8,13].

In contrast, the highly scripted process of preclinical and clinical development leading to product approval takes place almost entirely in industry, and virtually all the pharmaceutical products on the market today were developed and commercialized by for-profit companies [14–19], including 354/356 of the products studied by Cleary et al. [8].

Academic research is typically disseminated through publications in academic journals and presentations at scientific meetings as well as by educating students for the workforce with knowledge and know-how arising from scholarly activities, through academic-industry partnerships, or through formal and informal collaborations or consulting with industry [20–28]. Alternatively, inventions and patents arising from federally funded research can be licensed to industry for commercialization through the provisions of the “Patent and Trademark Law Amendments Act” of 1980, known as the Bayh-Dole Act [23,29–31].

The stated objectives of the Bayh-Dole Act are to “*…promote the utilization of inventions arising from federally supported research or development…*,” advance “…*the commercialization and public availability of inventions made in the United States by United States industry and labor…*,” and protect the public “*…against nonuse or unreasonable use of inventions*” [32]. By promoting commercialization of practical applications enabled by federally funded research, Bayh-Dole was designed to provide indirect returns to the public sector in the form of commercial products to address unmet public needs, create jobs, stimulate economic growth, and expand the tax base [23,33,34]. Additionally, by ceding the revenues from technology licenses to non-profit institutions incorporated in the public interest [35], Bayh-Dole positioned these institutions as proxies for the public sector in securing a direct return on public investment in research.

To achieve these objectives, the Bayh-Dole Act authorized public-sector institutions and small businesses to file patents on “subject inventions” made with federally funded research, required them to assess the potential value and, if appropriate, patent the invention, and established procedures for non-exclusive or exclusive licensing of the resulting patents to industry, while retaining selected government rights [8,29–31,36,37]. Bayh-Dole also authorized non-profit institutions to retain the proceeds from such licenses, providing that the proceeds are shared with the inventor and that institutional funds “*will be utilized for the support of scientific research or education*” [38].

There has been relatively little research on the terms of license agreements authorized by the Bayh-Dole Act. This is due in large measure to a scarcity of publicly available data on these licenses. While the Bayh-Dole Act requires extensive reporting of subject inventions, patents, and licenses, it also explicitly makes these reports confidential and not subject to public disclosure [38]. As a result, studies of the economic returns from academic licenses have focused primarily on data from selected, large universities [34,39], or aggregate data on sales of licensed products and revenues based on survey data collected by the Association of University Technology Managers (AUTM) (https://autm.net/surveys-and-tools/surveys) [40].

Some licenses involving public companies are reported to the Securities and Exchange Commission (SEC). The terms of license agreements between a public biopharmaceutical company and either an academic institution or another commercial firm is reported to the SEC if the company considers the license to be “material” to their valuation [41,42]. “Materiality” is legally defined as “*a substantial likelihood that the disclosure of the omitted fact would have been viewed by the reasonable investor as having significantly altered the ‘total mix’ of information*” [43]. In this standard, the materiality of a technology license is assessed only in relation to “*the significance of an item to users of a registrant’s financial statements*” (SEC, 1999) [42,44] rather than a uniform threshold. As a result, a license that may be judged material to the valuation of an emerging, public biotechnology company at the time of an Initial Public Offering (IPO) may not be material to more mature biotechnology or pharmaceutical companies where the “total mix” of information available to investors may be substantially greater.

License agreements reported to the SEC are available online or through the Freedom of Information Act (FOIA) (22 CFR Part 503) by filing an FOIA petition. The Bioscience database (www.bioscidb.com/) used in this analysis, as well as the earlier ReCap database (now Thomson Reuters) [1], were assembled through FOIA petitions. Using these datasets, Edwards described an average royalty rate for academic biotechnology licenses of 4% in the ReCap database through 2003 [1] and an average royalty rate of 3.22% in the Bioscience database from 2007–2013 [3]. Edwards also observed that the economic terms of academic licenses to for-profit firms are generally less favorable to academic institutions than comparable licenses between for-profit firms [1–5]. The present work extends these observations by asking whether differences in the economic returns from these licenses are associated with the intrinsic terms of these agreements. The residual disparity between the returns of academic and corporate licenses are discussed in the context of both the legal concept of a reasonable royalty rate and the role of technology licenses in providing a public return on government investments in biomedical science.

#### Benchmarks for returns on academic biotechnology licenses

The principle of a “reasonable royalty rate” arose from case law regarding the damages due to a patent holder from infringement. A reasonable royalty rate is defined as “*the amount which a prudent licensee who desired*, *as a business proposition*, *to obtain a license to manufacture and sell a particular article embodying the patented invention would have been willing to pay as a royalty and yet be able to make a reasonable profit and which amount would have been acceptable by a prudent patentee who was willing to grant a license*” [38,44–47]. In practice, “reasonableness” is determined by considering a “hypothetical negotiation” in which a variety of factors may influence the economic terms including exclusivity, the territories in which the licensee can practice the invention, the scope of protection offered by the licensed patents, and the commercial value of the embodiments enabled by the license.

Legal precedent recognizes that a reasonable royalty rate can be impacted by factors intrinsic to the license agreement as well as extrinsic factors that may impact the negotiated terms of a license. Among the factors identified in case law are the royalties paid for comparable patents or licenses, customary rates in comparable business, the licensor’s commitment to maintaining the patent, expected profitability of the product, degree of advantage offered by the patent, the portion of the profit that should be credited to the invention, as well as any ancillary commercial benefits provided to the licensee by the license [38]. In this context, it may be expected that the economic terms of license agreements may be reasonably impacted both by the terms of the license agreement and other attributes of academic, as opposed to corporate, research or licensing.

The returns from academic licenses may also be considered in the context of their role in public policy. Emerging economic theories have called attention to the foundational role of government in models of innovation in contextualizing government as a “lead investor” in innovation and government spending on basic or applied research as an “early-stage investment” [8,48–58]. As such, these theories posit that government, or the public sector it represents, could expect a return on investment commensurate with the risk of early-stage investment and comparable to the returns of analogous private sector investments [54,58–60]. Under the Bayh-Dole Act, the returns on technology licenses from academic institutions to industry represent the primary mechanism for providing the public sector with a return on investment.

NIH investment in basic and applied biomedical science plays an enabling role in pharmaceutical innovation and is frequently cited as an exemplar of government’s role as an “early investor” [8,51–54,60–62]. In this context, the economic terms from academic licenses of biotechnology can be meaningfully measured against the returns of comparable licenses between commercial firms.

## Materials and methods

### Dataset

The Bioscience dataset was provided by Bioscience Advisors (now Evaluate) (https://www.biosciadvisors.com/) and has been described previously [2–5]. Data are available at https://tinyurl.com/OnlineBioscienceDatabase1 or https://tinyurl.com/OnlineBioscienceDatabase2.

Economic metrics for each license included effective royalty rate on the first $500 million in sales, total deal size, and precommercial payments. The dataset categorizes the licensor and licensee of each license as “research institution,” “top pharma,” “mid-tier pharma,” “Japanese pharma,” “2013–2015 IPO,” “2013–2019 IPO,” “major biotech,” or “research institution.” For this analysis, licenses were classified as “academic” if the licensor is a “research institution” and corporate for all other licensors. Licensees were classified as “biotech” if the licensee is “2013–2015 IPO,” “2013–2019 IPO,” or not indicated and “pharma” for other licensees. Classifications were corrected to account for the status of the licensee at the date of the license agreement. All classifications were reviewed by two authors. The working dataset had three license classes: licenses between an academic institution and a biotechnology company (“academic-biotech”); licenses between two biotechnology companies (“corporate-biotech”); and licenses between a biotechnology company and larger pharmaceutical company (“corporate-pharma”). There were no licenses between academic institutions and larger pharmaceutical companies (“academic-pharma”) in the dataset.

The Bioscience dataset categorizes licenses as “exclusive,” “non-exclusive,” or “semi-exclusive.” For this analysis, “semi-exclusive” or “non-exclusive” licenses were classified as “non-exclusive.” The dataset also provides one or more descriptors of the “deal type” with a lexicon that includes “equity,” “research,” “development,” “co-development,” “co-promotion,” “distribution,” and other terms. For this analysis, deal type was classified as including equity, R&D (“research” or “development”), or “co-commercialization” (“co-development,” “co-promotion,” or “distribution”).

The dataset categorizes the development phase of drug products in the alliance as “discovery,” “lead molecule,” “preclinical,” “phase 1,” “phase 2,” “phase 3,” “filed,” or “approved.” For this analysis, we classified agreements by the most advanced phase and treated “filed” as “approved.” Licenses unrelated to drug development were excluded from this analysis.

### Analytical methods

Normality of data describing effective royalty rate, deal size, and precommercial payments was assessed by the Shapiro-Wilks test. Effective royalty rate, deal size, and precommercial payments were compared by Mann-Whitney. This analysis used non-parametric statistical methods, median univariate regression, and median multivariable regression due to the non-normality of the economic metric data.

Consistent with statements by the American Statistical Association [63], results are not described as “significant” or “non-significant,” but are described with p-values. To aid interpretation in the presence of multiple tests or regression coefficients, we indicate the Bonferroni correction for each analysis and the p-value equivalent to a threshold of 0.05. Analyses were performed in SPSS or Excel.

### Regression models

Median univariate regression was performed to estimate the median economic returns of academic and corporate licenses and the difference between these returns with 95% CI using Model I:

*I*. *Economic Metric = B*_*0*_ *+ B*_*1*_*License class + u*

where the Econometric Metric dependent variable is either the effective royalty rate, total deal size, or precommercial payments; the *License class* indicator variable was set to 0 for academic-biotech licenses and 1 for corporate-biotech licenses; and *u* is an independent and identically distributed error term with the assumption that the probability *u* is greater than zero is 50%. In this model, *B*_*0*_ estimates the median economic return for academic-biotech licenses and *B*_*1*_ estimates the difference in median economic return between the academic-biotech and corporate-biotech licenses. Analyses were performed separately for the effective royalty rate, deal size, and precommercial payments.

To analyze the license relationships from different perspectives, Model I (median univariate regression) was also performed with the indicator variable for *License class* set to 0 for corporate-biotech licenses and 1 for academic-biotech licenses such that *B*_*0*_ estimated the median economic return for corporate-biotech licenses and *B*_*1*_ the difference between corporate-biotech and academic-biotech licenses. Model I was also performed with the indicator variable *License class* set to 0 for corporate-biotech and 1 for corporate-pharma to compare corporate-biotech and corporate-pharma licenses, such that *B*_*1*_ estimates the difference in median economic return between corporate-biotech and corporate-pharma licenses.

A series of related median multivariable regressions was performed to consider the impact of different license characteristics on economic returns. The relationship between economic metrics and the development phase of products anticipated in the license for each of the three license types (academic-biotech, corporate-biotech, and corporate-pharma) was examined using Model II:

*II*. *Economic Metric = B*_*0*_ *+ B*_*s1*_*Phase2 + B*_*s2*_*Phase1 + B*_*s3*_*Preclincal + B*_*s4*_*LeadMolecule + u*

where indicator variables for each development phase were set to 1 for the most advanced product in the license agreement. In this model, *B*_*0*_ estimates the median economic return for the default phase (discovery) and *B*_*s1*_, *B*_*s2*_, *B*_*s3*_, and *B*_*s4*_ each estimates the difference in median economic returns between licenses at the discovery phase and their respective advanced development phases. Entries with phase 3 or approved drugs were excluded from this analysis due to insufficient data.

The relationship between economic metrics of academic and corporate licenses with control for the development phase of products anticipated in the license was examined using Model III:

*III*. *Economic Metric = B*_*0*_ *+ B*_*1*_*License class + B*_*s1*_*Phase2 + B*_*s2*_*Phase1 + B*_*s3*_*Preclincal + B*_*s4*_*LeadMolecule + u*

where an indicator variable for *License class* is set to 0 for academic-biotech and 1 for corporate-biotech. In this model, *B*_*0*_ estimates the median economic return for academic licenses, *B*_*1*_ estimates the difference in median economic returns for corporate licenses independent of development phase, and *B*_*s1*_, *B*_*s2*_, *B*_*s3*_, and *B*_*s4*_ each estimates the difference in median economic returns associated with development stage. Model III was also used to analyze a data subset that excluded license agreements with co-commercialization terms.

The relationship between economic metrics of academic and corporate licenses with controls for development phase of products anticipated in the license and license terms including exclusivity was examined using Model IV:

*IV*. *Economic Metric = B*_*0*_ *+ B*_*1*_*License class + B*_*s1*_*Phase2 + B*_*s2*_*Phase1 + B*_*s3*_*Preclincal + B*_*s4*_*LeadMolecule + B*_*e5*_*Exclusivity + u*

where the indicator variable for *License class* was set to 0 for academic-biotech licenses and 1 for corporate-biotech licenses, terms *B*_*s1*_, *B*_*s2*_, *B*_*s3*_, and *B*_*s4*_ are comparable to Model III, and the indicator variable for *Exclusivity* is set to 0 for exclusive and 1 for nonexclusive such that *B*_*e5*_ estimates the difference in median economic return for exclusive and nonexclusive licenses.

The relationship between economic metrics of academic and corporate licenses with controls for development phase of products anticipated in the license and deal type was examined using Model V:

*V*. *Economic Metric = B*_*0*_ *+ B*_*1*_*License class + B*_*s1*_*Phase2 + B*_*s2*_*Phase1 + B*_*s3*_*Preclincal + B*_*s4*_*LeadMolecule + B*_*d5*_*Deal Type + u*

where the indicator variable for *License class* was set to 0 for academic-biotech licenses and 1 for corporate-biotech licenses, terms *B*_*s1*_, *B*_*s2*_, *B*_*s3*_, and *B*_*s4*_ are comparable to Model III, and the indicator variable for deal type was set to 0 if the agreement does not include research or development and 1 if either is included such that *B*_*d*5_ estimates the difference in median economic return for licenses with and without research and development. Model V was also run with the indicator variable for *Deal type* set to 0 if there was no equity included and 1 if there was equity such that *B*_*d*5_ estimates the difference in median economic return for licenses with and without equity.

The relationship between economic metrics, development phase of products anticipated in the license, and license terms including co-commercialization was examined using Model VI:

*VI*. *Economic Metric = B*_*0*_ *+ B*_*s1*_*Phase2 + B*_*s2*_*Phase1 + B*_*s3*_*Preclincal + B*_*s4*_*LeadMolecule + B*_*c5*_*Co-commercialization + u*

where the indicator variable for *License class* was removed, terms *B*_*s1*_, *B*_*s2*_, *B*_*s3*_, and *B*_*s4*_ are comparable to Model III, and the indicator variable for *Co-commercialization* is set to 0 if the deal type does not include co-commercialization terms (co-development, co-promotion, or distribution) and 1 if any of these terms were present, such that *B*_*c5*_ estimates the difference in median economic return for licenses with and without co-commercialization.

## Results

### Data description

**Fig 1:**
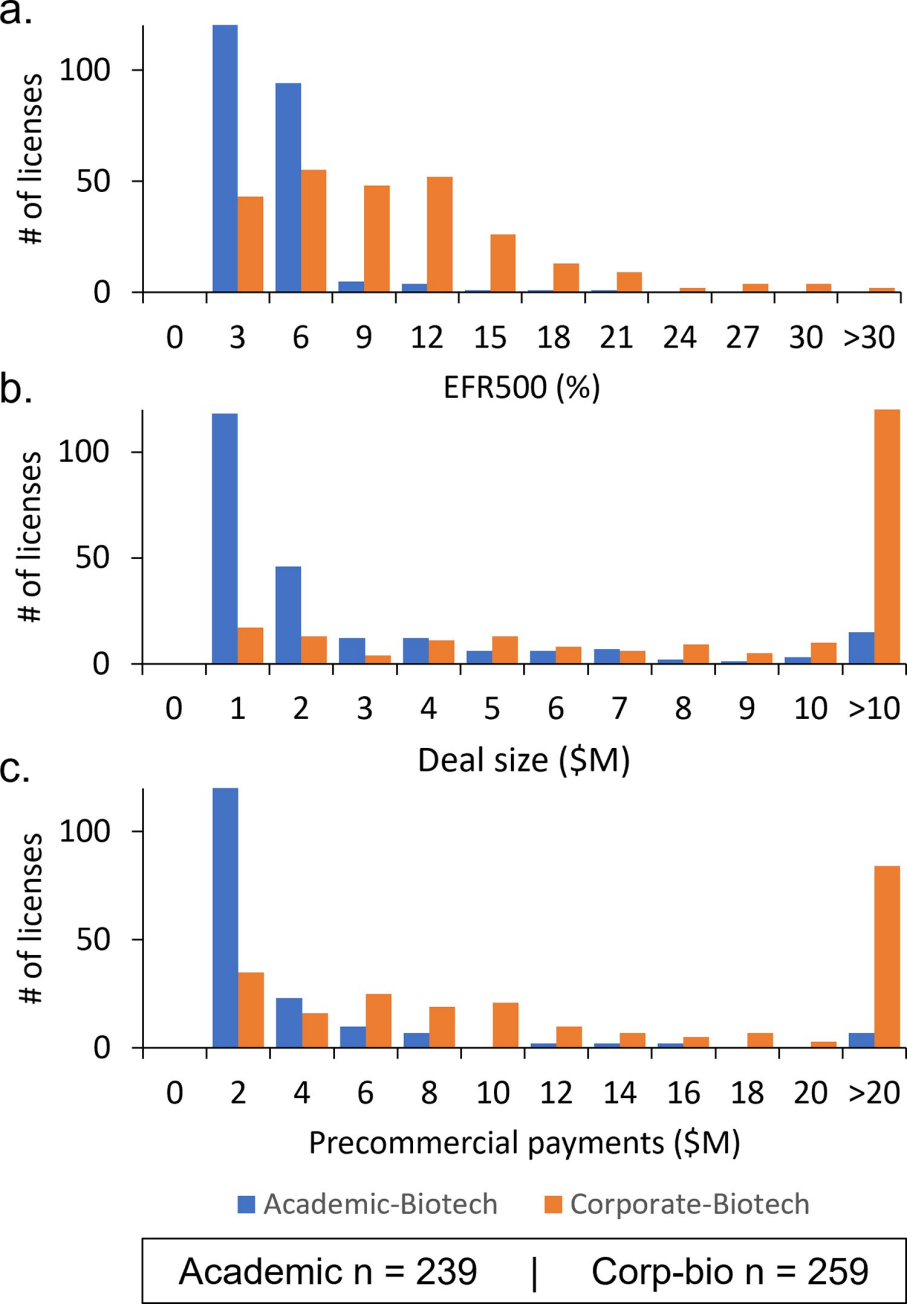
Economic returns from academic and corporate licenses to biotechnology companies. (a.) Effective royalty rate on $500M in net sales; (b.) Total reported deal size; (c.) Total precommercial payments. Analysis excludes licenses to pharmaceutical companies.

This Bioscience database includes 1,379 licenses of biomedical technologies executed from 1987–2019 and obtained through FOIA petitions with the SEC. Fig 1 shows the distribution of the effective royalty rate, deal size, and precommercial payments for academic-biotech and corporate-biotech licenses. The effective royalty rate, deal size, and precommercial payments were not normally distributed (Shapiro-Wilks test, p<0.001). Consequently, this analysis used non-parametric statistical methods including median univariate and multivariable regression.

### Comparing academic and corporate licenses

The Bioscience database had 239 academic-biotech licenses, 259 corporate-biotech licenses, and 657 corporate-pharma licenses. There were no academic-pharma licenses, which is consistent with the fact that agreements with academic institutions are less likely to be considered material to the valuation of large, revenue-driven pharmaceutical companies than they are to research-stage biotechnology companies.

The median economic returns from academic licenses were lower than from corporate licenses (i.e., all corporate licenses including corporate-biotech and corporate-pharma) for effective royalty rate (3% versus 8%, Mann Whitney p<0.001), deal size ($0.9M versus $31.0M, Mann Whitney p<0.001), and precommercial payments ($1.1M versus $25.4M, Mann Whitney p<0.001). This analysis has a Bonferroni correction of 3, with p = 0.016 equivalent to a p = 0.05 threshold.

Corporate-pharma licenses had greater median deal size ($40.1M versus $17.3M, p<0.001) and precommercial payments ($32.9M versus $10.0M, p<0.001) than corporate-biotech licenses, but similar median effective royalty rate (8.6% versus 7.6%, p = 0.072) (Model I, Table 1). With a Bonferroni correction of 3 in this analysis, p = 0.016 is equivalent to a threshold of p = 0.05. To eliminate ascertainment bias from the absence of academic-pharma licenses, subsequent analyses compared academic-biotech and corporate-biotech licenses.

**Table 1:** Economic returns for biotechnology licenses by licensor and licensee.

	Effective Royalty Rate	Deal Size	Precommercial Payments
	Coefficient (95% CI), p		
**Academic vs. corporate licenses** **(to biotech firms)** ^**1**^			
Intercept	3.0 (2.5,3.5), <0.001	0.9 (0.1,1.7), 0.024	1.1 (0.1,2.0), 0.023
Academic/Corporate (0,1)	5.0 (4.3,5.7), <0.001	16.3 (15.2,17.4), <0.001	9.1 (7.8,10.3), <0.001
**Corporate vs. academic licenses** **(to biotech firms)** ^**2**^			
Intercept	8.0 (7.5,8.5), <0.001	17.3 (16.5,18.0), <0.001	10.1 (9.3,10.9), <0.001
Corporate/Academic (0,1)	-5.0 (-5.7,-4.3), <0.001	-16.3 (-17.4,-15.2), <0.001	-9.1 (-10.3,-7.8), <0.001
**Corporate licenses to biotech vs.** **pharma firms** ^**3**^			
Intercept	7.6 (6.7,8.5), <0.001	17.3 (10.2,24.3), <0.001	10.0 (4.6,15.4), <0.001
Biotech/Pharma (0,1)	1.0 (-0.1,2.1), 0.072	22.8 (14.5,31.0), <0.001	22.9 (16.6,29.2), <0.001

^1^ Univariate median regression Model I with licensor indicator variable academic = 0, corporate = 1. Note that all academic licenses are with biotech licensees. In this model, the intercept represents the median return from academic licenses.

^2^ Univariate median regression Model I with licensor indicator variable corporate = 0, academic = 1. In this model, the intercept represents the median return from corporate licenses.

^3^ Univariate median regression Model I with licensee indicator variable biotech = 0, pharma = 1. This analysis has a Bonferroni correction of 3, with p = 0.016 equivalent to a p = 0.05 threshold.

Univariate median regression (Model I) shows that academic-biotech licenses had lower median effective royalty rate (3% versus 8%, p<0.001), median deal size ($0.9M versus $17.2M, p<0.001), and median precommercial payment ($1.1M versus $10.2M, p<0.001) (Table 1). This analysis has a Bonferroni correction of 3, with p = 0.016 equivalent to a p = 0.05 threshold. Thus, the disparity in economic returns between academic and corporate licenses remains substantial after eliminating the bias from the absence of academic-pharma licenses.

### Impact of development phase on academic and corporate license returns

S1 Table shows the development phase of academic-biotech and corporate-biotech licenses. Academic licenses had fewer products in phased trials (23% versus 45%), fewer products in phase 3 (1% versus 12%), and no approved products. The Chi Square test of association between development phase and academic versus corporate licenses (excluding phase 3 and approved licenses) had a p-value of <0.001.

**Fig 2:**
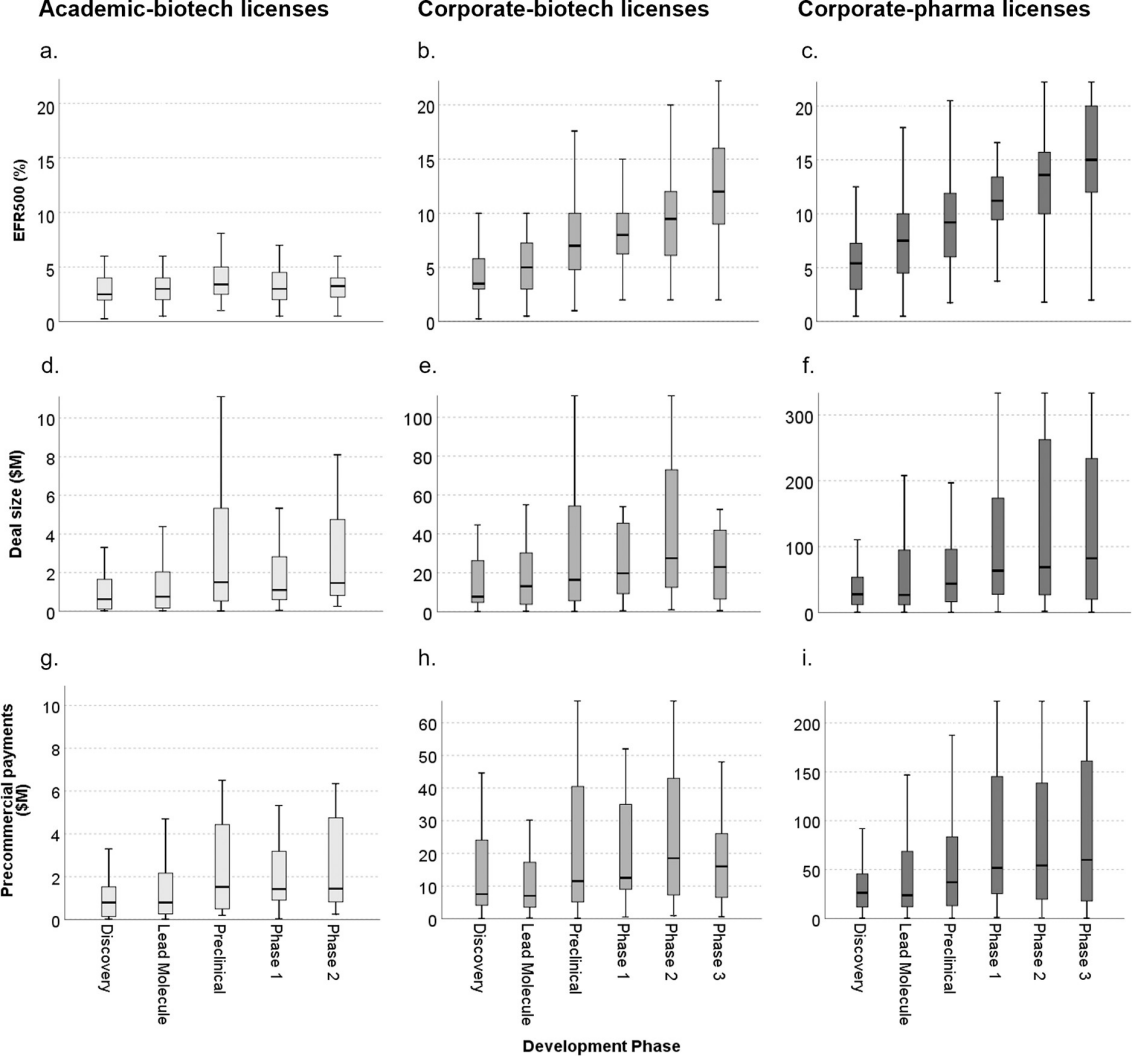
Economic returns from academic-biotech, corporate-biotech, and corporate-pharma licenses by development phase of lead product. Effective royalty rate on $500M in net sales for (a.) Academic-biotech licenses; (b.) Corporate-biotech licenses; and (c.) Corporate-pharma licenses. Deal size for (d.) Academic-biotech licenses; (e.) Corporate-biotech licenses; and (f.) Corporate-pharma licenses. Precommercial payments for (g.) Academic-biotech licenses; (h.) Corporate-biotech licenses; and (i.) Corporate-pharma licenses. Development phase is defined as the development phase of the most advanced product (discovery, lead molecule, preclinical, phase 1, phase 2, phase 3, or approved). Phase 3 trials are not included in data describing academic licenses due to insufficient data. Note differences in the scale for the deal size and precommercial payments of different license classes.

The relationship between economic returns and development phase (Fig 2, S2–S4 Tables) was quantified by multivariate median regression (Model II). This analysis has a Bonferroni correction of 12, with p = 0.0042 equivalent to a p = 0.05 threshold. The economic returns of academic licenses did not increase with development advancing through phase 2. In contrast, for corporate-biotech licenses, economic returns increased with development beyond the discovery phase, with effective royalty rate increasing by 1.5% to 6% (p<0.001 beyond lead molecule), median deal size increasing by $4.4M to $19.8M (p>0.0042), and median precommercial payments increasing by $3.8M to $12.8M (p>0.0042), though deal size and precommercial payments exhibited broad variation and higher p-values (Table 2). For corporate-pharma licenses, economic returns increased with development phase, with effective royalty rate increasing by 2.1% to 8.2% (p<0.001 beyond lead molecule), median deal size increasing by $17M for preclinical to $45.1M for phase 2 (p<0.001 beyond preclinical), and median precommercial payments increasing by $10.8M to $30.3M (p<0.001 for phase 2 only) (Table 2). Unlike corporate licenses, academic licenses with products more advanced in development do not provide greater economic returns. Median and Interquartile Range (IQR) of the effective royalty rate, deal size, and precommercial payments for academic-biotech, corporate-biotech, and corporate-pharma licenses by development phase are shown in S2–S4 Tables.

**Table 2:** Economic returns for biotechnology licenses by license class and development phase.

License class	N	Effective Royalty Rate	Deal Size	Precommercial Payments
Development phase				
		Coefficient (95% CI), p		
**Academic-Biotech**	** **	** **	** **	** **
Intercept		2.5 (1.9,3.1), <0.001	0.6 (0.1,1.1), 0.013	0.8 (0.2,1.4), 0.013
Phase 3	2	n/a	n/a	n/a
Phase 2	20	1.0 (-0.2,2.2), 0.091	0.9 (-0.2,1.9), 0.097	0.7 (-0.6,1.9), 0.31
Phase 1	33	0.5 (-0.5,1.5), 0.31	0.5 (-0.3,1.3), 0.25	0.6 (-0.4,1.6), 0.22
Preclinical	61	0.9 (0.1,1.7), 0.03	0.9 (0.2,1.6), 0.014	0.7 (-0.1,1.6), 0.087
Lead Molecule	57	0.5 (-0.3,1.3), 0.24	0.1 (-0.6,0.8), 0.72	-0.1 (-0.9,0.8), 0.84
Discovery^1^	63	n/a	n/a	n/a
**Corporate-Biotech**		** **	** **	
Intercept		3.5 (2.2,4.8), <0.001	7.7 (-2.9,18.3), 0.16	7.7 (-1.1,16.5), 0.085
Phase 3	25	n/a	n/a	n/a
Phase 2	43	6.0 (4.1,7.8), <0.001	19.8 (4.9,34.7), 0.01	12.8 (0.4,25.2), 0.043
Phase 1	23	4.5 (2.3,6.7), <0.001	12.1 (-6.1,30.2), 0.19	4.8 (-10.1,19.7), 0.53
Preclinical	39	3.5 (1.6,5.4), <0.001	8.7 (-6.6,24.0), 0.26	3.8 (-8.8,16.4), 0.55
Lead Molecule	32	1.5 (-0.5,3.5), 0.14	4.4 (-13.5,22.3), 0.63	-0.7 (-15.6,14.2), 0.93
Discovery^1^	42	n/a	n/a	n/a
**Corporate-Pharma**		** **	** **	
Intercept		5.4 (4.7,6.1), <0.001	27.6 (19.4,35.7), <0.001	26.3 (19,33.5), <0.001
Phase 3	84	n/a	n/a	n/a
Phase 2	79	8.2 (6.8,9.6), <0.001	45.1 (29.7,60.4), <0.001	30.3 (16.6,43.9), <0.001
Phase 1	41	5.8 (4,7.6), <0.001	41.5 (21.6,61.3), <0.001	25.6 (7.9,43.2), 0.005
Preclinical	112	4.0 (2.8,5.2), <0.001	17 (3.2,30.7), 0.015	10.8 (-1.5,23.0), 0.086
Lead Molecule	66	2.1 (0.6,3.6), 0.005	-1.9 (-18.7,15.0), 0.83	-3.3 (-18.3,11.8), 0.67
Discovery^1^	203	n/a	n/a	n/a

Multivariate regression (Model II) with indicator variables for development phase through phase 2. Licenses with phase 3 or approved products are excluded.

n/a—no indicator variable included in analysis for phase 3 or discovery.

^1^ Baseline for development phase data. This analysis has a Bonferroni correction of 12, with p = 0.0042 equivalent to a p = 0.05 threshold.

To assess whether the more advanced development phase of products in corporate alliances could account for their greater economic returns, multivariate median regression (Model III) was performed with an indicator variable for academic-biotech versus corporate-biotech licenses and indicator variables for development phase through phase 2 (Table 3). In this model, economic returns of academic licenses were still lower than corporate licenses, with a difference in median effective royalty rate of 3% (p<0.001), median deal size of $15.8M (p<0.001), and median precommercial payments of $9.0M (p<0.001). This analysis has a Bonferroni correction of 3, with p = 0.016 equivalent to a p = 0.05 threshold. The difference in median effective royalty rate was reduced from 5% to 3%, below the 95% CI of regression performed without the development phase control, but the differences in median deal size and precommercial payments were unchanged. Thus, the less advanced development phase of products in academic licenses accounts for a portion of the disparity in royalties, but not differences in deal size or precommercial payments.

**Table 3:** Economic returns for biotechnology licenses by licensee and development phase.

	Effective Royalty Rate	Deal Size	Precommercial Payments
	Coefficient (95% CI), p		
**Intercept**	2.0 (1.4,2.6), <0.001	0.4 (-0.4,1.3), 0.32	0.6 (-.5,1.7), 0.27
**Licensor: Academic/Corporate (0,1)**	3.0 (2.4,3.6), <0.001	15.8 (14.9,16.6), <0.001	9.0 (8.0,10.0), <0.001
**Phase 2**	3.0 (2.1,3.9), <0.001	4.3 (3.0,5.6), <0.001	1.8 (.2,3.4), 0.028
**Phase 1**	1.5 (.6,2.4), 0.001	1.2 (-0.1,2.5), 0.079	1.0 (-.7,2.6), 0.25
**Preclinical**	1.8 (1.0,2.6), <0.001	1.1 (-.02,2.2), 0.054	0.9 (-.4,2.3), 0.18
**Lead Molecule**	1.0 (.2,1.8), 0.015	0.3 (-0.9,1.4), 0.64	0.1 (-1.4,1.5), 0.94
**Discovery** ^**1**^	n/a	n/a	n/a

Multivariate median regression (Model III) with indicator variable for academic-biotech (0) or corporate-biotech (1) licenses and indicator variables for development phases from discovery through phase 2. n/a—no indicator variable included in analysis for discovery. ^1^ Baseline for development phase data. This analysis has a Bonferroni correction of 3, with p = 0.016 equivalent to a p = 0.05 threshold.

### Impact of deal terms on academic and corporate license returns

To assess how exclusivity impacts economic returns, multivariate median regression (Model IV) was performed with the indicator variables for academic-biotech versus corporate-biotech licenses, development phases through phase 2, and an indicator variable for exclusivity (Table 4). Nonexclusive licenses had lower economic returns than exclusive licenses, with a difference in median effective royalty rate of -1.6% (p = 0.003), median deal size of $-0.6M (p = 0.38), and median precommercial payment of $-0.9M (p = 0.25). The difference in economic returns between academic and corporate licenses remained within the 95% CI calculated without the exclusivity control. This analysis has a Bonferroni correction of 6, with p = 0.008 equivalent to a p = 0.05 threshold. Thus, the lower economic returns of academic licenses are not related to differences in the value of exclusive versus nonexclusive licenses.

**Table 4:** Median regression coefficients by licensor, most advanced development phase and deal terms.

Regression Model	Effective Royalty Rate	Deal Size	Precommercial Payments
	Coefficient (95% CI), p		
**a. Exclusive/non-exclusive**			
**Intercept**	2.1 (1.4,2.8), <0.001	0.6 (-0.3,1.5), 0.17	0.9 (-0.2,2.1), 0.10
**Licensor: Academic/Corporate (0,1)**	3.6 (3.0,4.2), <0.001	15.3 (14.4,16.1), <0.001	8.6 (7.6,9.5), <0.001
**Development Phase**	Not shown		
**Exclusive/Non-Exclusive (0,1)**	-1.6 (-2.7,-0.5), 0.003	-0.6 (-1.9,0.7), 0.38	-0.9 (-2.5,0.6), 0.25
**b. R&D**			
**Intercept**	2.0 (1.2,2.8), <0.001	0.2 (-0.8,1.2), 0.73	0.4 (-0.9,1.8), 0.54
**Licensor: Academic/Corporate (0,1)**	3.2 (2.5,3.8), <0.001	16.2 (15.3,17.1), <0.001	8.8 (7.8,9.9), <0.001
**Development Phase**	Not shown		
**R&D (0,1)**	0.4 (-0.3,1.0), 0.28	0.5 (-0.4,1.3), 0.28	0.4 (-0.7,1.4), 0.48
**c. Equity**			
**Intercept**	2.0 (1.3,2.7), <0.001	0.3 (-0.6,1.1), 0.54	0.3 (-0.9,1.4), 0.65
**Licensor: Academic/Corporate (0,1)**	3.0 (2.4,3.6), <0.001	16.1 (15.3,16.9), <0.001	9.4 (8.4,10.4), <0.001
**Development Phase**	Not shown		
**Equity (0,1)**	0.7 (-0.1,1.5), 0.078	0.8 (-0.2,1.8), 0.10	0.8 (-0.4,2.0), 0.18
**d. Co-commercialization licenses excluded**			
**Intercept**	2.0 (1.4,2.6), <0.001	0.3 (-0.6,1.2), 0.53	0.6 (-0.3,1.5), 0.18
**Licensor: Academic/Corporate (0,1)**	3.0 (2.4,3.6), <0.001	11.4 (10.4,12.3), <0.001	7.6 (6.8,8.4), <0.001
**Development Phase**	Not shown		

Multivariate median regression with licensor indicator variable academic-biotech (0) or corporate-biotech (1); indicator variables for development phase from discovery through phase 2. (a.) With indicator variable with exclusive/nonexclusive (0/1) (Model IV); (b.) With indicator variable for R&D (0/1) (Model V); (c.) With indicator variable for equity (0/1) (Model V); (d.) With co-commercialization licenses excluded (Model III). This analysis has a Bonferroni correction of 3, with p = 0.016 equivalent to a p = 0.05 threshold.

Licenses with R&D comprise 69% (n = 164) of academic-biotech licenses compared to 42% (n = 108) of corporate-biotech licenses. Licenses with equity comprise 22% (n = 52) of academic-biotech agreements and 18% (n = 47) of corporate-biotech agreements. Licenses with co-commercialization terms (co-development, co-promotion, or distribution) comprise 0.8% (n = 2) of academic-biotech licenses compared to 19% of corporate-biotech licenses (n = 50).

To assess the impact of deal types including R&D or equity on the lower economic returns of academic licenses, multivariate median regression was performed with an indicator variable for academic-biotech versus corporate-biotech licenses, indicator variables for development phases through phase 2, and an indicator variable for deal type (Model V). With the indicator variable for R&D, the difference in median between academic and corporate licenses for effective royalty rate was 3.2% (p<0.001), for deal size was $16.2M (p<0.001), and for precommercial payments was $8.8M (p<0.001) (Table 4). Each value is within the 95% CI calculated without the R&D control. With the indicator variable for equity, the difference in median between academic and corporate licenses for effective royalty rate was 3.0% (p<0.001), for deal size was $16.1M (p<0.001), and for precommercial payments was $9.4M (p<0.001) (Table 4). Each value is within the 95% CI calculated without the equity control. This analysis has a Bonferroni correction of 3, with p = 0.016 equivalent to a p = 0.05 threshold. Thus, the lower economic returns of academic licenses are not related to the inclusion of either R&D or equity in the deal type.

To assess the effect of co-commercialization terms on economic returns, multivariate regression was performed with indicator variables for development phase and co-commercialization (Model VI). Co-commercialization is associated with greater effective royalty rate (difference in median of 4%, p<0.001), deal size (difference in median of $41M, p<0.001), and precommercial payments (difference in median of $37.3M, p<0.001) (S5 Table). This analysis has a Bonferroni correction of 3, with p = 0.016 equivalent to a p = 0.05 threshold.

To assess whether the scarcity of academic licenses involving co-commercialization could account for lower economic returns, multivariate median regression was run on a subset of licenses that excluded co-commercialization with indicator variables for License class and development phase (Model III). In this analysis (Table 4), the difference in median effective royalty rate (3%, p<0.001) was within the 95% CI of the same analysis on the full dataset (Table 3), but the difference in median deal size ($11.4M, p<0.001) and precommercial payments ($7.6M, p<0.001) were below the 95% CI of the same analysis on the full dataset (Table 3). These data indicate that the lower deal size and precommercial payments associated with academic licenses, but not royalties, are partially related to the absence of co-commercialization in academic licenses.

## Discussion

This empirical analysis confirms the observation that the economic returns to academic institutions from licenses of biotechnologies arising from federally funded research are substantially lower than those associated with comparable corporate licenses. While the absolute value of the economic returns is influenced by the development stage of products, whether the licensee was a biotechnology or large pharmaceutical company, and whether the license agreement involved co-commercialization, the disparity between academic and corporate licenses is largely independent of these factors.

This analysis was potentially biased by the absence of licenses between academic institutions and large pharmaceutical companies in the Bioscience dataset. Even after eliminating corporate licenses to large pharmaceutical companies, corporate licenses had a 2–3 times higher effective royalty rate than academic licenses as well as 10–20 times higher deal size and precommercial payments.

This analysis demonstrated a relationship between the development phase of the most advanced products in the license agreement and the effective royalty rate of corporate, but not academic, licenses. Coupled with the more advanced clinical stage of products associated with corporate licenses, this relationship partially accounts for the higher royalty rates of corporate licenses. Even after controlling for clinical phase, however, the effective royalty rate of corporate licenses was 2 times higher than that of academic licenses. There was also a relationship between the inclusion of co-commercialization in the license agreement and both deal size and precommercial payments. However, when repeating the analysis on a subset of license agreements that did not include co-development, the deal size and precommercial payments of corporate licenses were 10 times higher than academic licenses. These analyses suggest that academic licenses are undervalued relative to comparable corporate licenses even after controlling for the intrinsic elements of the license agreements.

The “reasonableness” of undervaluing academic licenses can be considered in the context of the legal framework of a reasonable royalty rate. This standard recognizes that factors external to the license agreement may contribute to its negotiated value [38,47]. Previous studies have identified that several aspects of academic licensing might contribute to the lower value of academic licenses.

First, while the Bayh-Dole Act provides for academic institutions to enter exclusive licenses for federally funded technologies, the degree of exclusivity that can be provided by academic institutions may be limited. An exclusive license agreement between two corporate firms may include not only patent rights, but also exclusive access to trade secrets, know how, and materials. In contrast, academic investigators are ultimately expected to disseminate their results to the scientific community through publications in academic journals and presentations at scientific meetings, and are commonly required to share research materials, methods, and data by funding agencies or scientific journals [31,64,65]. Moreover, workforce training is a core mission of many academic institutions and the students or post-doctoral fellows who are engaged in the licensed research may be hired by companies explicitly for their related knowledge and skills. In addition, many faculty engage in conversations, collaborations, or consulting with companies outside of the university’s formal technology transfer process, which can further erode the exclusivity of a technology license [66]. These dynamics may compromise the portion of any future profit that would be credited to the invention and may reasonably reduce the value of the license.

Second, there is widespread concern about the reproducibility of academic research. An unpublished study from Amgen reportedly found that only 6/53 (11%) “landmark” cancer studies could be confirmed [67]. Similar concerns have arisen from other studies [68–71] and have been largely confirmed by the prospectively designed Reproducibility Project [72]. While a prudent licensee would be expected to perform diligence on the validity of technologies when determining a reasonable price, others may discount the value of the license due to concerns about the reproducibility of academic findings, thus depressing the median value of the licenses in the dataset.

Third, several studies have questioned the experience, business acumen, and negotiating skills of university technology transfer professionals [73,74]. While the (in)experience of technology transfer offices has been cited as contributing to “overpricing” of technologies and the inefficiency of university licensing [24], we are not aware of any evidence that the business experience of technology transfer offices contributes lower economic returns.

Fourth, academic alliances with biotechnology companies may often lead to vertical alliance networks, in which the technology licensed to a biotechnology firm is subsequently licensed to a larger pharmaceutical company for development and commercialization [1,75]. Such licensing can raise the fixed cost of the resulting product [76]. Similarly, the open, often-competitive nature of academic research often leads to multiple patents covering separate aspects of a technology. In such cases, a company may need to secure multiple licenses from different institutions, leading to “royalty stacking” [77] or may even make it impossible for the licensee to fully acquire freedom to operate. This is the so-called “tragedy of the anticommons” [78]. This dynamic may limit the price that a corporate licensee would be reasonably willing to pay for a technology.

Conversely, there is also evidence that biotechnology companies may derive substantial benefits from licensing academic technologies that extend beyond the technology itself. Studies suggest that a biotechnology firm’s relationship with “star” scientists and institutions provide scientific capital in the form of academic networks [26,79–81] and a workforce of university graduates [82–87]. These companies have been shown to bring more products to market, create more jobs, and achieve a higher market capitalization [81,88,89]. Such benefits might increase the negotiated value of academic licenses to commercial firms.

Applying the legal framework of a reasonable royalty rate, it may be argued that such factors might reasonably impact the negotiated terms of license agreements, leading to lower effective royalty rates, deal sizes, and precommercial payments for academic licenses compared to corporate licenses. Considering the returns from academic licenses as a mechanism for providing a social return on public sector investments in pharmaceutical innovation, however, suggests a different perspective.

Lazonick, Mazzucato, and others have argued that, as an early investor in biomedical science and pharmaceutical innovation, government (or the public sector it represents) should expect a return on investment commensurate with comparable investments by industry [54,58–60]. This study demonstrates explicitly that the economic terms of academic licenses fail to meet this standard and that the returns on academic licenses are significantly lower than those of corporate licenses with comparable terms.

It should be emphasized that the primary objective of federal funding for academic biomedical research is not to generate economic activity but rather to “enhance health, lengthen life, and reduce illness and disability” [90,91]. A full assessment of the returns on government investments in biomedical research would need to address the social returns on these investments for many stakeholders including the economic returns to academic institutions, improved population health, increased scientific capital and technological capability, job creation, economic growth, and taxes [92–94] as well as the prices paid for these products in the marketplace [95].

### Limitations of this study

First, the dataset is limited to licenses that were considered “material” to the for-profit firms and reported to the SEC. Thus, it does not include licenses between non-profit entities or privately held companies, licenses between academic institutions and large pharmaceutical companies, for whom academic licenses are not typically “material,” or agreements between faculty and companies that are not intermediated by the university [96].

Second, this analysis may underestimate the direct returns from technology licenses in the form of equities or downstream royalties. While equity represents only a small amount (<4%) of total licensing revenue, the royalties from successful products (or sale of prospective royalty streams for approved products) can be many times greater than the value of prescribed payments for a small number of licenses [39]. Conversely, this analysis may overestimate the value of milestone payments or other incentives that may not be realized.

## Conclusion

This empirical study demonstrates that the economic returns associated with biotechnology licenses from academic institutions are systematically lower than licenses between commercial firms and that this difference is only partially accounted for by the clinical stage of the licensed product, co-commercialization, or other intrinsic terms of the license agreements. Further research is required to examine the relationship between factors extrinsic and the negotiated economic returns from these licenses and the broad, social return on public sector investments in early-stage, pharmaceutical innovation.

## Supporting information

S1 Table Median and IQR of Effective Royalty Rate (EFR) for academic-biotech, corporate-biotech, and corporate-pharma licenses by development phase. (DOCX)

S2 Table Median and IQR of deal size for academic-biotech, corporate-biotech, and corporate-pharma licenses by development phase. (DOCX)

S3 Table Median and IQR of precommercial payments for academic-biotech, corporate-biotech, and corporate-pharma licenses by development phase. (DOCX)

S4 Table Multivariate median regression (Model VI) on the effect of deal terms for co-commercialization and development phase on economic returns. ^1^ Multivariate regression model VI with indicator variable for licenses with deal terms for commercialization (1/0). Co-commercialization terms included co-development, co-promotion, and distribution. With a Bonferroni correction of 3, p = 0.016 is equivalent to a threshold of p = 0.05.
(DOCX)

S5 Table Definitions of variables. ^1^ Definitions from Mark Edwards, Bioscience Advisors.
(DOCX)
