# Supplementary material for: Comparing the economic terms of biotechnology licenses from academic institutions with those between commercial firms
Source: PLoS One. 2023 Mar 31;18(3):e0283887. doi: 10.1371/journal.pone.0283887 (PMC10065281; doi:10.1371/journal.pone.0283887)
Supplement: S1 Table — (DOCX) [file pone.0283887.s001.docx]

| **S1 Table.** Median and IQR of Effective Royalty Rate (EFR) for academic-biotech, corporate-biotech, and corporate-pharma licenses by development phase. | | | | | | | | | | |
| --- | --- | --- | --- | --- | --- | --- | --- | --- | --- | --- |
|  |  |  | |  | |  | |  | |  |
|  | **academic-biotech** | | |  | | **corporate-biotech** | |  | | **corporate-pharma** |
| **Development phase** | **N** | **MEDIAN (IQR) EFR (%)** | **N** | | **MEDIAN (IQR) EFR (%)** | | **N** | | **MEDIAN (IQR) EFR (%)** | |
| Discovery | 63 | 2.5 (2.0,4.0) | 42 | | 3.5 (3.0,5.7) | | 203 | | 5.4 (3.0,7.3) | |
| Lead Molecule | 57 | 3 (2.0,4.0) | 32 | | 5.0 (3.0,7.3) | | 66 | | 7.5 (4.5,10.0) | |
| Preclinical | 61 | 3.4 (2.5,5) | 39 | | 7 (4.8,10.0) | | 112 | | 9.2 (6.0,11.9) | |
| Phase 1 | 33 | 3 (2.0,4.5) | 23 | | 8.0 (6.3,10.0) | | 41 | | 11.2 (9.5,13.4) | |
| Phase 2 | 20 | 3.3 (2.4,4.0) | 43 | | 9.5 (6.1,12.0) | | 79 | | 13.6 (10.0,15.7) | |
| Phase 3 | 2 | 2.0 (n/a) | 25 | | 12.0 (9.0,16.0) | | 84 | | 15.0 (12.0,20.0) | |
| Filed | 0 | N/A | 6 | | 12.0 (N/A) | | 19 | | 15.0 (10.7,20.5) | |
| Approved | 0 | N/A (N/A) | 30 | | 12.7 (10.0,16.4) | | 34 | | 11.0 (8.0,19.5) | |
| **All** | **236** | **3.0 (2.0,4.0)** | **240** | | **7.8 (4.5,12.0)** | | **638** | | **8.6 (5.0,13.3)** | |
