# Supplementary material for: Comparing the economic terms of biotechnology licenses from academic institutions with those between commercial firms
Source: PLoS One. 2023 Mar 31;18(3):e0283887. doi: 10.1371/journal.pone.0283887 (PMC10065281; doi:10.1371/journal.pone.0283887)
Supplement: S2 Table — (DOCX) [file pone.0283887.s002.docx]

| **S2 Table.** Median and IQR of deal size for academic-biotech, corporate-biotech, and corporate-pharma licenses by development phase. | | | | | | |
| --- | --- | --- | --- | --- | --- | --- |
|  |  |  |  |  |  |  |
|  | **academic-biotech** | |  | **corporate-biotech** |  | **corporate-pharma** |
| **Development phase** | **N** | **MEDIAN (IQR)  Deal Size ($M)** | **N** | **MEDIAN (IQR) Deal Size ($M)** | **N** | **MEDIAN (IQR) Deal Size ($M)** |
| Discovery | 63 | 0.6 (0.1,1.7) | 42 | 7.7 (4.8,26.1) | 203 | 27.6 (11.9,53.4) |
| Lead Molecule | 57 | 0.8 (0.2,2.0) | 32 | 13.1 (4.0,29.8) | 66 | 26.6 (11.9,91.6) |
| Preclinical | 61 | 1.5 (0.5,5.3) | 39 | 16.4 (5.6,54.4) | 112 | 43.8 (16.5,95.8) |
| Phase 1 | 33 | 1.1 (0.6,2.8) | 23 | 19.8 (9.3,45.5) | 41 | 63.5 (28.6,168.5) |
| Phase 2 | 20 | 1.5 (0.8,4.4) | 43 | 27.5 (12.5,73.0) | 79 | 68.8 (27.4,254.7) |
| Phase 3 | 2 | 0.8 (N/A) | 25 | 23.0 (6.5,41.9) | 84 | 82.3 (21.3,232.5) |
| Filed | 0 | N/A (N/A) | 6 | 35.8 (N/A) | 19 | 55.0 (36.0,164.5) |
| Approved | 0 | N/A (N/A) | 30 | 26.4 (8.6,130.0) | 34 | 29.0 (7.5,134.0) |
| **All** | **236** | **0.9 (0.3,2.4)** | **240** | **17.3 (5.2,46.9)** | **638** | **40.0 (15.2,100.0)** |
