# Supplementary material for: Comparing the economic terms of biotechnology licenses from academic institutions with those between commercial firms
Source: PLoS One. 2023 Mar 31;18(3):e0283887. doi: 10.1371/journal.pone.0283887 (PMC10065281; doi:10.1371/journal.pone.0283887)
Supplement: S3 Table — (DOCX) [file pone.0283887.s003.docx]

| **S3 Table.** Median and IQR of precommercial payments for academic-biotech, corporate-biotech, and corporate-pharma licenses by development phase. | | | | | | | | | | |
| --- | --- | --- | --- | --- | --- | --- | --- | --- | --- | --- |
|  |  |  | |  | |  | |  | |  |
|  | **academic-biotech** | | |  | | **corporate-biotech** | |  | | **corporate-pharma** |
| **Development phase** | **N** | **MEDIAN (IQR)  Precommercial Payment ($M)** | **N** | | **MEDIAN (IQR) Precommercial Payment ($M)** | | **N** | | **MEDIAN (IQR) Precommercial Payment ($M)** | |
| Discovery | 63 | 0.8 (0.1,1.5) | 42 | | 7.5 (4.4,23.3) | | 203 | | 26.3 (11.7,45.5) | |
| Lead Molecule | 57 | 0.8 (0.3,2.0) | 32 | | 7.0 (3.5,17.3) | | 66 | | 23.8 (12.0,68.1) | |
| Preclinical | 61 | 1.5 (0.5,4.4) | 39 | | 11.5 (5.2,40.5) | | 112 | | 37.0 (13.1,83.5) | |
| Phase 1 | 33 | 1.4 (0.9,3.2) | 23 | | 12.5 (9.0,35.0) | | 41 | | 51.8 (25.4,145.3) | |
| Phase 2 | 20 | 1.4 (0.8,4.8) | 43 | | 18.5 (7.4,42.5) | | 79 | | 54.3 (19.9,135.3) | |
| Phase 3 | 2 | 1.5 (N/A) | 25 | | 16.0 (6.5,26.0) | | 84 | | 60.0 (18.0,161.1) | |
| Filed | 0 | N/A (N/A) | 6 | | 23.5 (N/A) | | 19 | | 40.0 (20.0,125.5) | |
| Approved | 0 | N/A (N/A) | 30 | | 14.0 (3.5,63.8) | | 34 | | 14.0 (3.8,138.0) | |
| **All** | **236** | **1.1 (0.4,2.4)** | **240** | | **10.1 (4.7,30.0)** | | **638** | | **32.9 (13.5,87.5)** | |
