# Supplementary material for: Comparing the economic terms of biotechnology licenses from academic institutions with those between commercial firms
Source: PLoS One. 2023 Mar 31;18(3):e0283887. doi: 10.1371/journal.pone.0283887 (PMC10065281; doi:10.1371/journal.pone.0283887)
Supplement: S4 Table — 1 Multivariate regression model VI with indicator variable for licenses with deal terms for commercialization (1/0). Co-commercialization terms included co-development, co-promotion, and distribution. With a Bonferroni correction of 3, p = 0.016 is equivalent to a threshold of p = 0.05. (DOCX) [file pone.0283887.s004.docx]

| **S4 Table.** Multivariate median regression (Model VI) on the effect of deal terms for co-commercialization and development phase on economic returns. | | | |
| --- | --- | --- | --- |
|  |  |  |  |
| **Regression model^1^** | **EFR500** | **Deal size** | **Precommercial payments** |
|  | **Coefficient (95% CI), p** | | |
| **Intercept** | 3.0 (2.4,3.6), <0.001 | 1.2 (-0.6,2.9), 0.20 | 1.7 (-0.2,3.5), 0.088 |
| **Phase 2** | 3.2 (2.2,4.2), <0.001 | 12.9 (10,15.7), <0.001 | 7.4 (4.4,10.3), <0.001 |
| **Phase 1** | 1.8 (0.8,2.8), 0.001 | 0.9 (-2.1,3.8), 0.57 | 2.0 (-1.1,5.1), 0.20 |
| **Preclinical** | 1.0 (0.1,1.9), 0.022 | 2.4 (-0.1,4.8), 0.063 | 1.6 (-1,4.2), 0.23 |
| **Lead Molecule** | 1.0 (0.1,1.9), 0.027 | 0.1 (-2.5,2.7), 0.95 | -0.4 (-3.2,2.4), 0.77 |
| **Discovery** | n/a | n/a | n/a |
| **Co-commercialization** | 4.0 (2.8,5.2), <0.001 | 41.0 (37.5,44.5), <0.001 | 37.3 (33.8,40.7), <0.001 |
| ^1^ Multivariate regression model VI with indicator variable for licenses with deal terms for commercialization (1/0). Co-commercialization terms included co-development, co-promotion, and distribution. With a Bonferroni correction of 3, p=0.016 is equivalent to a threshold of p=0.05. | | | |
