# Supplementary material for: Comparing the economic terms of biotechnology licenses from academic institutions with those between commercial firms
Source: PLoS One. 2023 Mar 31;18(3):e0283887. doi: 10.1371/journal.pone.0283887 (PMC10065281; doi:10.1371/journal.pone.0283887)
Supplement: S5 Table — 1 Definitions from Mark Edwards, Bioscience Advisors. (DOCX) [file pone.0283887.s005.docx]

| **S5 Table.** Definitions of variables. | |  |  |
| --- | --- | --- | --- |
|  | |  |  |
| Variable | Definition^1^ | | |
| EFR | The Effective Royalty Rate (EFR) owed by licensee to licensor in the event that annual net sales reach $500M | | |
| Deal size | A summation of all upfront, R&D, and milestone payments, including any equity or loan amounts, to be paid to the licensor | | |
| Total Precommercial payments | The sum of upfront, equity, sponsored R&D, loans, Dev/Reg and additional product and/or indication milestones to be paid to the licensor through launch in all jurisdictions | | |
| Co-Development | The licensor performs unfunded development activities post-signing | | |
| Co-Promotion | Two legal entities with the joint right to sell a single product | | |
| Distribution | Right to access supply and sell product | | |
| Development | The licensor performs funded development activities post-signing | | |
| Research | The licensor performs funded research activities post-signing | | |
| Equity | Purchase of a share of ownership of one legal entity by another legal entity | | |
| ^1^ Definitions from Mark Edwards, Bioscience Advisors. | | |  |
